# Improving the timeliness of meticillin-resistant *Staphylococcus aureus* antimicrobial decolonization therapy administration: a descriptive account^☆^

**DOI:** 10.1016/j.jhin.2014.01.004

**Published:** 2014-03

**Authors:** H.L. Brooks, J. Hodson, S.J. Richardson, L. Stezhka, M.J. Gill, J.J. Coleman

**Affiliations:** a University Hospitals Birmingham NHS Foundation Trust, Edgbaston, Birmingham, UK; b College of Medical and Dental Sciences, University of Birmingham, Birmingham, UK

**Keywords:** Antibiotic administration, Clinical Decision Support, Computerized Provider Order Entry, Intervention, MRSA decolonization

## Abstract

**Background:**

It is important to ensure that the timely administration of appropriate antimicrobial decolonization therapy occurs when patients are identified as meticillin-resistant *Staphylococcus aureus* (MRSA)-colonized. Computerized Provider Order Entry (CPOE) with embedded Clinical Decision Support (CDS) may help to facilitate this.

**Aim:**

To investigate changes in the average time from patient admission to administration of MRSA decolonization antimicrobial therapy in the context of various national and local infection control interventions, including the use of CPOE.

**Methods:**

Data concerning the time of admission and of administration of patients' first MRSA decolonization antimicrobials were extracted from a locally developed CPOE system (Prescribing Investigation and Communications System: PICS) which was introduced at a large university teaching hospital in the UK in 1998. Data were extracted retrospectively from January 2006 to March 2012.

**Findings:**

A variety of relevant local and national interventions occurred from 2006 to 2012. Notably, the automatic charting of MRSA decolonization antimicrobial therapy was introduced in December 2007. There was a significant decline of 15.0% per year (95% confidence interval: 11.1–18.7%; *P* < 0.001) in the time taken from admission to administration of MRSA decolonization antimicrobial therapy during the study period.

**Conclusions:**

Numerous factors may have contributed to the observed reductions in the time from admission to administration of MRSA decolonization antimicrobials, including the implementation of specific features within a CPOE system. By rapidly attending to positive MRSA colonizations there is decreased potential for MRSA to spread, which may help to reduce the prevalence of MRSA colonizations within hospitals and improve patient outcomes.

## Introduction

Healthcare-associated infections (HCAIs) such as those due to meticillin-resistant *Staphylococcus aureus* (MRSA) are problematic in hospitals worldwide.^1^ MRSA eradication is a priority in UK hospitals and reductions in the prevalence of MRSA bacteraemias (i.e. bloodstream infections) may be attributed to a number of factors, including routine screening for MRSA colonization (i.e. asymptomatic carriers), transmission-based precautions (e.g. use of antibacterial handwash), antimicrobial stewardship (e.g. prescribing antibiotics only when necessary to prevent new antibiotic resistance) and patient isolation.^2–6^ It is important to promote the timely detection of MRSA colonization in patients and administration of appropriate antimicrobial drugs (e.g. mupirocin, chlorhexidine) for decolonization, which may help to reduce the opportunity for MRSA to spread within hospitals.

The use of Computerized Provider Order Entry (CPOE) systems with embedded Clinical Decision Support (CDS) may help to ensure that appropriate treatment responses rapidly follow the detection of HCAIs such as MRSA, by improving workflow and efficiency. Following the implementation of a CPOE system, Chapman *et al.* observed a non-significant reduction from 131 to 125 min in the time from admission to administration of antibiotics in a neonatal intensive care unit (ICU).^7^ Furthermore, Panosh *et al.* found a significant reduction from 3.18 to 2.00 h in the time from ordering to administration of antimicrobial drugs after CPOE had been introduced into their university teaching hospital.^8^ Other studies have suggested that CPOE has helped to decrease both staff work time and rates of MRSA in hospitals following the implementation of the automatic ordering of MRSA surveillance cultures by the CPOE system and improved rates of patient isolation following the provision of electronic alerts upon the identification of multidrug-resistant bacteria.^6,9^ CPOE systems with embedded CDS may eliminate the need to manually complete certain stages in the process from patient admission to monitoring for and treating antimicrobial infection. In the present study, we investigated the time taken from patient admission to administration of MRSA decolonization antimicrobial therapy in the context of various local and national policy changes and in relation to relevant changes to our CPOE system.

## Methods

### Setting and study population

**Figure 1:**
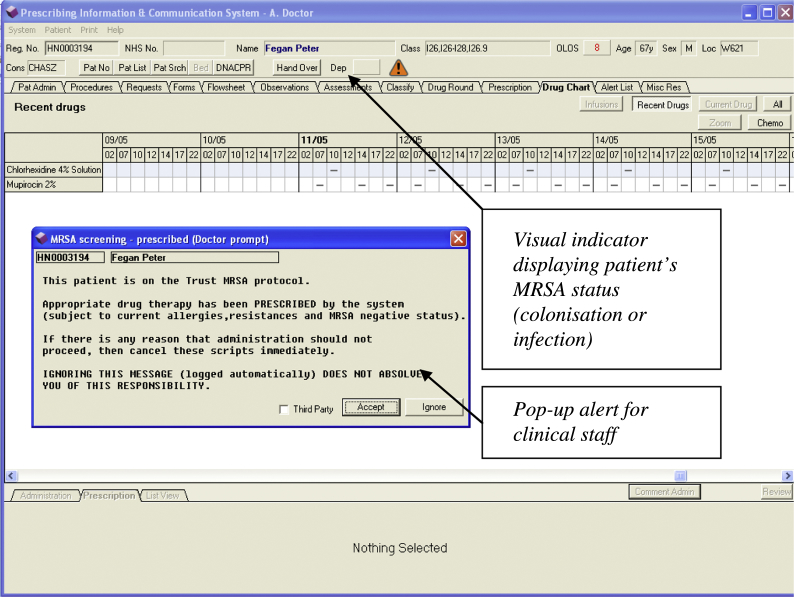
Screen shot example of patient record when patient is MRSA positive.

This work was carried out in a large National Health Service (NHS) hospital in the UK. The hospital has prioritized infection prevention and control, and specifically MRSA decolonization.^10^ This has resulted in strict MRSA-screening procedures and prompt treatment of positive cases.^11^ The hospital uses a locally developed CPOE system – Prescribing Investigation and Communications System (PICS) – which is embedded with CDS. PICS was co-designed by clinical and technical experts within the hospital and is in use throughout all (∼1200) inpatient beds and for all prescribing, except for some chemotherapy regimens. PICS was first installed on the renal unit in 1998.^12^ By 2006, more than 50% of the beds in the hospital were live on PICS and the roll-out was fully completed in 2008. PICS now covers all general and specialist medical and surgical specialties in the hospital (excluding obstetric, paediatric, and mental health patients, which are treated in other organizations). Importantly, for the purpose of this study, upon the receipt of a new positive MRSA laboratory result from any sample tested by the microbiology department, PICS alerts users to the presence of MRSA. This is notified by a pop-up alert to clinical staff and then continuously represented by a visual indicator within the individual patient record, an example of which is displayed in Figure 1. Furthermore, the system records the date and time at which each laboratory sample is identified as MRSA positive, and the time at which associated decolonizing antimicrobial drugs are prescribed and administered.

### Intervention

MRSA response capabilities were first introduced into PICS in 2005. Since then, governmental guidelines and hospital policies have impacted upon the nature of MRSA screening and decolonization processes within the hospital (e.g. the timings of screenings throughout inpatient spells), infection prevention and control protocols (e.g. mandatory handwashing), and features within PICS relating to MRSA.^13–18^ For example, in November 2007 it became possible for PICS to automatically populate the record for MRSA antimicrobial decolonization therapy following the identification of positive MRSA swabs. Whereas the majority of prescriptions will be configured automatically by the system, it remains possible for doctors to manually prescribe MRSA-decolonizing antimicrobials prior to the receipt of a positive laboratory result. Other factors, such as the use of the polymerase chain reaction (PCR) approach to detect MRSA between January 2008 and January 2011, or the implementation of chromogenic agar for MRSA screening to allow for faster identification of MRSA colonization, may also have impacted upon the timeliness of the administration of MRSA antimicrobials. A summary of relevant local and national interventions is presented in Table I.

### Data capture

This study used data from January 2006 to March 2012 concerning the dates and times that patients (i) were first admitted to hospital, and (ii) were first administered MRSA antimicrobial therapy. Data were collected retrospectively. From 2006 onwards large units within the hospital, such as General Medicine, started using PICS. We therefore extracted data from 2006 onwards as after this point the majority of data could be captured and reported. For the purpose of this study we analysed data relating to the first identified case of MRSA colonization during the first hospital admission for each patient only (growth of MRSA from screening swabs or PCR detection from screens). This was due to the system automatically prompting for (before December 2007) or prescribing (from December 2007) MRSA-decolonizing antimicrobials on any subsequent admissions for those patients who were colonized during their first admission. As a result, the process from admission to administration of MRSA decolonization antimicrobial therapy is different for patients during subsequent inpatient spells. There were too few cases of readmission data to perform a comparative analysis of these data in this study. Furthermore, we excluded MRSA bacteraemia cases. This was due to the limited number of bacteraemia cases and differences in the time taken to administer MRSA decolonization antimicrobial therapy as a result of differences in the time needed for colonization and bacteraemia samples to be cultured in the laboratory. This meant that it was not possible to combine the respective cases for analysis.

### Analysis

Due to the amount of skew in the distribution, the time from admission to administration of MRSA decolonization therapy was log_10_-transformed prior to analysis. The resulting variable was then set as the dependent variable in a regression model. For each admission, the time, in years, from the commencement of the study was calculated for each patient and included as a continuous covariate in this model.

The coefficient from the regression model could then be interpreted as the yearly change in the average log_10_-transformed time from admission to administration of MRSA-decolonization therapy. Hence, the anti-log_10_ of this value would represent the multiplicative yearly change in the time from admission to administration of MRSA-decolonization therapy. This was then further converted to a percentage change for ease of interpretation.

All analyses were performed using IBM SPSS 19 (IBM Corp., Armonk, NY, USA), with *P* < 0.05 considered statistically significant.

## Results

Data were obtained for a total of 1403 cases. For 1316 (94%) of these cases the time of patient admission and the time of the first administration of antimicrobials for MRSA decolonization were recorded.

**Figure 2:**
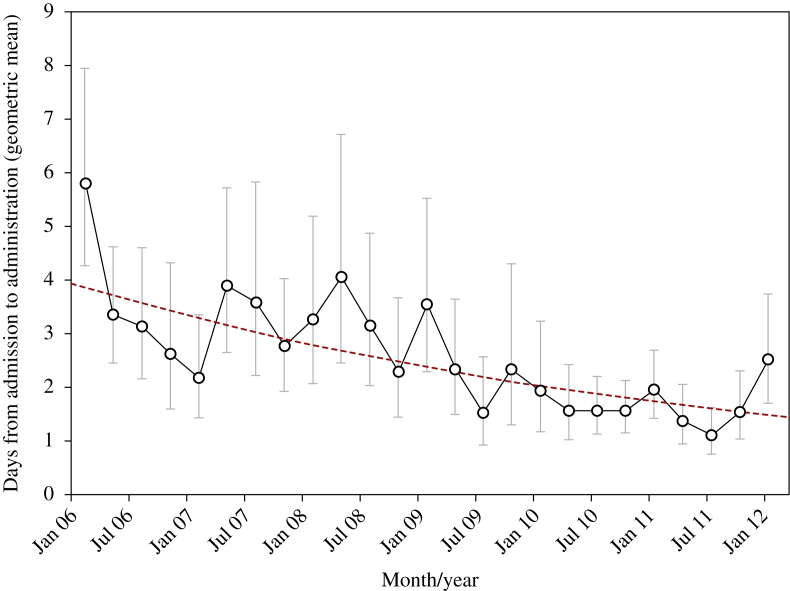
Geometric mean time from admission to administration of MRSA decolonization therapy for patients with positive MRSA colonization swabs.

**Figure 3:**
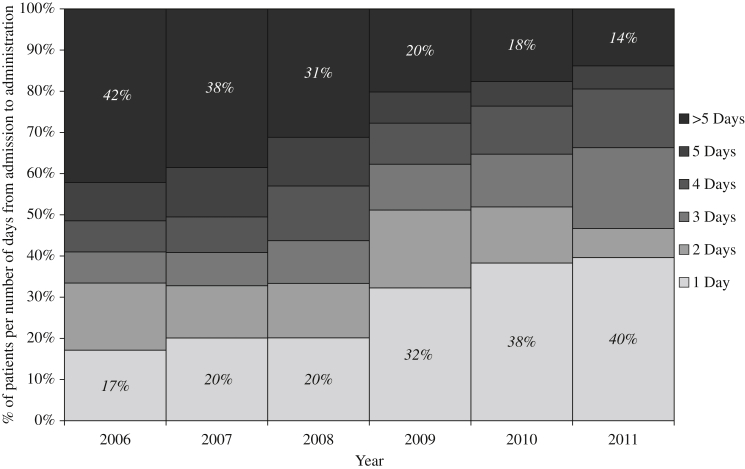
Number of days from admission to the administration of MRSA decolonization therapy.

As Figure 2 displays, the time from admission to first administration of MRSA-decolonizing antimicrobial therapy was found to be in significant decline over the period of the study (*P* < 0.001). The coefficient from the model estimates a reduction in this time of 15.0% per year (95% confidence interval: 11.1–18.7%) over the course of the study. In line with this, the number of patients who had MRSA-decolonizing antimicrobial therapy administered within one, two, three, four or five days of admission increased from 2006 to 2011 (Figure 3). The percentage of patients treated within one day rose by 22.4% from 17.3% of 179 cases in 2006 to 39.7% of 300 cases in 2011. Conversely, the number of patients whose administration took more than five days decreased from 41.9% in 2006 to 13.7% in 2011.

From 2007 to 2008 there was a 7.2% increase in the number of patients treated within five days: from 61.6% of 164 cases to 68.8% of 144 cases. This coincided with the introduction of automatic charting of MRSA-decolonizing antimicrobial therapy and use of the PCR detection method, as well as changes to the screening procedures and treatment regimens within the hospital. In 2009 it became mandatory for all patients to be swabbed upon admission, and from 2008 to 2009 a 12.2% increase (from 20.1% of 144 cases to 32.3% of 133 cases) was observed in the number of patients treated within one day of admission. Finally, from 2009 to 2010, there was a 6.0% increase (from 32.3% of 133 cases to 38.3% of 329 cases) in the number of patients treated within one day, which coincided with updates to the Health and Social Care Act 2008 and the implementation of chromogenic agar for MRSA screening.^18^

## Discussion

Across the period of the study, we observed significant reductions in the length of time from admission to administration of MRSA decolonization antimicrobial therapy. The time decreased by an average of 15.0% per year, from 4.0 days in January 2006 to 1.4 days in March 2012. Overall, the number of patient days, per year, spent waiting for treatment has decreased substantially from 838 in 2006 to 367 in 2011. As various national and local interventions related to MRSA prevention occurred in close temporal proximity, it is impossible to attribute causality to individual factors regarding the observed reductions. Instead, the increased speed at which MRSA decolonization antimicrobials are administered following admission to hospital may be attributed to a number of factors. These include changes to local infection prevention and control procedures in response to national policies and improvements to technology, including the introduction of MRSA-specific features within the CPOE system in use at this hospital.^14,16^

In addition to the legal requirement that hospitals must have systems in place to reduce the prevalence of HCAIs and to promptly identify those patients with MRSA, the introduction of MRSA response capabilities into PICS in 2005 was intended to trigger an institution-wide awareness of the importance of MRSA decolonization.^14^ Subsequently, this should have resulted in greater effort to reduce time to treatment in MRSA-colonized patients and may have led to other infection prevention and control procedures unrelated to the system. For example, the percentage of patients whose decolonization antimicrobial therapy was administered within one day of admission has increased over time (e.g. 17.3% of the 179 positive MRSA colonizations identified in 2006 were treated within one day; by 2011 this proportion had risen to 39.7% of the 326 positive cases), whereas the percentage of drugs administered more than five days after admission has decreased (e.g. 41.9% in 2006 to 13.7% in 2011).

The automatic charting feature was introduced into our CPOE system in December 2007, which effectively eliminates the time that would previously have been taken to raise doctors' awareness of a positive laboratory result and for them to prescribe the appropriate decolonizing antimicrobials. This time-saving may provide some explanation for the 7.2% increase in the number of patients administered decolonization antimicrobials within five days of admission from 2007 to 2008. Despite the implementation of automatic charting in December 2007 and the introduction of the faster PCR laboratory method in January 2008, there was no change in the percentage of patients administered decolonization antimicrobials within one day of admission in the first year (20.1% in both years). Thus, in 2008 automatic charting helped curtail the number of slowly processed cases (i.e. ≥5 days from admission to administration) but did not facilitate an increase in more rapid administration of decolonization antimicrobials of ≤2 days from admission to administration. This may be accounted for partly by changes to the patient groups who were screened for MRSA (i.e. critical care patients and admissions from other hospitals or nursing homes were automatically prescribed MRSA decolonization therapy upon admission) who would therefore have been excluded from our data set from January 2008 onwards. Nonetheless, once the feature was embedded, introducing the automatic charting feature may have contributed to the observed decrease in the overall geometric mean time from admission to administration over this study period.

Furthermore, the mandatory screening of specific patients upon admission was introduced in 2008, and of all patients upon admission in 2009. It is interesting that these interventions coincided with further increases in the percentage of patients administered MRSA decolonization antimicrobial therapy within one day of admission – from 2008 to 2009 there was a 12.2% increase and from 2009 to 2010 a further increase of 6.0%. In 2010, the use of chromogenic agar for MRSA screening was implemented, which may have contributed to the greater proportion of screens being processed within one day. The increase in the absolute number of MRSA screens led to a need for the microbiology department to process the swabs more rapidly using the Kiestra laboratory automation system. Greater efficiency may have contributed to a greater proportion of swabs being processed on the day of them being taken. However, it is possible that other factors, such as a heightened awareness of the importance of MRSA decolonization following the publication of governmental reports (e.g. Health and Social Care Act 2008), also contributed to an increase in the timeliness of the administration of MRSA-decolonizing antimicrobials.^16^

Any CPOE intervention requires both training and rigid application. All hospital staff required to use PICS receive formal training, and in this case rigidity was ensured by mandatory and automatic functions being implemented into PICS. It may still be possible to produce an incorrect output through incorrect user interaction, as has been discussed in previous papers.^19,20^ We hope that through our system we minimize the risk of workarounds and variation in related behaviours, meaning that the system can effectively perform its role; in this case reducing the time taken to administer MRSA antimicrobials.

The average time from admission to administration at the end of the study period was a little more than one day. Using current laboratory methods, swabs must be processed overnight in the laboratory. This means that, unless all laboratory results are processed on the same day as the patient is admitted, it may not be possible to reduce the time from admission to administration much further.

It must be noted that these data were extracted from a locally developed CPOE system at one NHS hospital and therefore may not be generalizable to other institutions, nor where other systems are in use. Furthermore, as previously stated, we were unable to utilize data from the few cases of MRSA bacteraemia that were presented at the hospital over the study period, nor compare data from patients' first hospital admission to subsequent hospital admissions. It was unfortunate that we were unable to investigate the statistical significance of individual interventions; however, the relative contribution of each intervention may only be small and lead to an overall significant decrease in the time taken from admission to administration of decolonization antimicrobial therapy in MRSA-colonized patients. Despite the hospital's policy to isolate patients colonized with MRSA, we were unable to examine the impact of patient isolation in this study. It was not possible to quantify retrospective data about the rates of MRSA isolation in this hospital, particularly as the hospital underwent a change in site in May 2011, with which came a change to the availability of single-bed patient rooms within the hospital. Furthermore, this was a single-site study and therefore no comparative data were available. Finally, due to differences in definitions and record accuracy/completeness, we were unable to compare the timeliness of administration of MRSA decolonization antimicrobial therapy via traditional paper-based prescribing (pre-implementation) with electronic-based prescribing.

Given the potential uses of CPOE and CDS embedded within these systems in helping to prevent MRSA, future work could consider the effectiveness of CPOE in improving the rapid identification and treatment of other multidrug-resistant bacteria such as extended-spectrum-producing beta-lactamase Enterobacteriaceae, or other infections, such as disease caused by *Clostridium difficile*. Furthermore, it would be interesting to investigate how CPOE can be used to improve the treatment of MRSA in different departments separately (e.g. intensive therapy unit, clinical decision unit), as well as investigating staff perceptions of the use of CPOE in controlling MRSA in hospitals.

In conclusion, it is important to continue to improve the timeliness of MRSA detection and its treatment. In our hospital, we observed reductions in the time taken from patient admission to administration of MRSA decolonization antimicrobial therapy. Such reductions may be attributed to a variety of factors, including national policies and local interventions. Improvements in information technology within the hospital, such as the automatic ordering of MRSA decolonization antimicrobial therapy within our CPOE system, may also help to improve the timeliness of administration of MRSA-decolonizing antimicrobials. CPOE with embedded CDS may support other infection prevention and control methods by improving efficiency in the process from patient admission to administration of decolonization antimicrobial therapy. Therefore alongside potential time-savings the opportunity for MRSA to spread is more limited, which may contribute to reducing the prevalence of MRSA in hospitals and improving patient outcomes.

## Figures and Tables

**Table I:** Summary of national (●) and local (○) interventions related to MRSA decolonization^a^

Date	Intervention
June 2005	● *Saving Lives: a delivery programme to reduce healthcare associated infection including MRSA*: series launched to raise awareness of MRSA and evidence-based guidelines for best practice regarding treatment and prevention.^13^
September 2005	○ Microbiology department reports positive MRSA results to PICS. ○ Patient MRSA status becomes visible on PICS via coloured symbols (positive, mupirocin resistant; positive, mupirocin sensitive; previously positive). ○ Clinical Decision Support (CDS) proposes prescription of MRSA decolonization therapy and an alert is generated for doctors to authorize the prescription. ○ Upon authorization, nurses are alerted to administer drugs.
October 2006	● Health Act 2006: DoH requirement for hospitals to have systems in place to minimize HCAIs.^14^
June 2007	● DoH recommends MRSA screening for preoperative patients, emergency admissions to critical care, dialysis patients, previously positive patients, elective surgical patients, oncology/chemotherapy patients, and patients admitted from high-risk settings.^15^
November 2007	○ New weekly drug administration therapy regimen (‘5 days on, 2 days off’) updated in PICS.
December 2007	○ CDS automatically populates MRSA decolonization therapy except in the presence of drug allergies. ○ A function is available to override automatic prescribing if clinically appropriate. ○ If ‘no longer positive’ flag received via the patient record, the rule ends the prescription of MRSA drugs at the end of 7-day cycle.
January 2008	○ Changes made to patient admission screens to identify MRSA risk (previous admission to UHB or other hospital in past 6 months, or admitted from residential/care home) and these patients are automatically prescribed MRSA decolonization therapy. In critical care, all patients are screened for MRSA and the treatment protocol is automatically started. ○ PCR detection of MRSA screens from acute surgical unit started. MRSA PCRs (nasal swabs: GeneOhm™ StaphSR; BD Diagnostics, Oxford, UK) run twice daily, Monday–Friday or morning only on Saturdays and Bank Holidays. Results released into PICS at about 12:00 and 16:00. ○ Implementation of Kiestra laboratory automation system to process more MRSA screens more rapidly.
July 2008	● Health & Social Care Act 2008.^16^
March 2009	○ Long-stay swab implemented (swab alert on PICS every 28 days unless on MRSA protocol).
April 2009	○All patients swabbed upon admission.
June 2009	● National Audit Office Publication: *Reducing HCAIs in hospitals in England.*^17^
April 2010	● Update to Health and Social Care Act 2008: patients and staff must be protected against HCAIs. Suitable systems must be in place to detect, prevent and control HCAIs, treat those infected and maintain relevant premises, equipment and materials.^18^
December 2010	○ Implementation of chromogenic agar for MRSA screening. Allows presumptive MRSA to be identified after overnight incubation.
January 2011	○ PCR detection of MRSA screens from acute surgical unit stopped.
2012	No relevant interventions.

MRSA, meticillin-resistant *Staphylococcus aureus*; PICS, Prescribing Investigation and Communications System; DoH, Department of Health; HCAI, healthcare-associated infection; UHB, University Hospitals Birmingham; PCR, polymerase chain reaction.

a Some differences apply in the critical care (intensive therapy unit) department only.
